# Dietary patterns in an elderly population and their relation with bone mineral density: the Rotterdam Study

**DOI:** 10.1007/s00394-016-1297-7

**Published:** 2016-08-24

**Authors:** Ester A. L. de Jonge, Fernando Rivadeneira, Nicole S. Erler, Albert Hofman, André G. Uitterlinden, Oscar H. Franco, Jessica C. Kiefte-de Jong

**Affiliations:** 1 Department of Epidemiology, Erasmus MC, Dr. Molewaterplein 50, PO box 2040, 3000 Rotterdam, The Netherlands; 2 Department of Internal Medicine, Erasmus MC, PO box 2040, 3000 Rotterdam, The Netherlands; 3 Department of Biostatistics, Erasmus MC, PO box 2040, 3000 Rotterdam, The Netherlands; 4 Department of Global Public Health, Leiden University College, PO box 13228, 2501 The Hague, The Netherlands; 5 Department of Epidemiology, Harvard T.H. Chan School of Public Health, Boston, MA USA

**Keywords:** Dietary patterns, Bone mineral density, Principal component analysis, Overall diet, Body weight

## Abstract

**Purpose:**

Our aim was to identify dietary patterns that are associated with bone mineral density (BMD) against a background of relatively high dairy intake in elderly Dutch subjects.

**Methods:**

Participants were 55 years of age and older (*n* = 5144) who were enrolled in The Rotterdam Study, a population-based prospective cohort study. Baseline intake of 28 pre-defined food groups was determined using a validated food frequency questionnaire. Dietary patterns were identified using principal component analysis. BMD was measured using dual-energy X-ray absorptiometry at baseline and at three subsequent visits (between 1993 and 2004). Linear mixed modelling was used to longitudinally analyse associations of adherence to each pattern with repeatedly measured BMD (both in *Z* scores).

**Results:**

After adjustment for confounders, two dietary patterns were associated with high BMD: a “Traditional” pattern, characterized by high intake of potatoes, meat and fat (*β* = 0.06; 95 % CI 0.03, 0.09) and a “Health conscious” pattern, characterized by high intake of fruits, vegetables, poultry and fish (*β* = 0.06; 95 % CI 0.04, 0.08). The “Processed” pattern, characterized by high intake of processed meat and alcohol, was associated with low BMD (*β* = −0.03; 95 % CI −0.06, −0.01). Associations of adherence to the “Health conscious” and “Processed” pattern with BMD were independent of body weight and height, whereas the association between adherence to the “Traditional” pattern with BMD was not.

**Conclusions:**

Against a background of high dairy intake and independent of anthropometrics, a “Health conscious” dietary pattern may have benefits for BMD, whereas a “Processed” dietary pattern may pose a risk for low BMD.

**Electronic supplementary material:**

The online version of this article (doi:10.1007/s00394-016-1297-7) contains supplementary material, which is available to authorized users.

## Introduction

In the recent decades, the role of individual nutrients such as calcium and vitamin D in healthy bone remodelling of elderly has been studied extensively [1]. However, people do not eat isolated nutrients but, rather, a combination of different foods. Hence, nutritional research is shifting from a traditional approach of investigating the effects of single nutrients (e.g. calcium or vitamin D) and foods (e.g. dairy products) to a more holistic approach investigating overall dietary patterns. Studying dietary patterns might help us to identify potential additive, synergistic or antagonistic effects between components of the full diet that may affect bone mineral density (BMD) [2]. Also, cumulative effects of a combination of nutrients on BMD might be easier to identify than the effect of a single nutrient, which might be too small to detect [3].

Dietary patterns differ between populations and depend on cultural habits and food availability. Identifying the dietary patterns associated with high or low BMD in different populations might help to identify common combinations of food groups or food products that are important for bone health. The current food-based dietary guidelines for maintaining BMD in populations where low BMD is prevalent recommend sufficient intake of calcium and vitamin D [4], mainly via dairy consumption. However, evidence regarding the effect of dietary patterns on BMD in populations with high dairy intake is scarce. Since average dairy consumption in the Netherlands is relatively high (ca. 350 g dairy/day [5] including milk, yoghurt and cheese), studying the full dietary patterns of the Dutch elderly can provide insights into the relationship between overall diet composition and BMD against a background of high dairy intake.

Mechanical loading of the weight-bearing bones is an important determinant of BMD [6]. Weight loss might decrease mechanical loading, whereas weight gain might increase mechanical loading [7, 8]. In response to a decrease or increase in mechanical loading, altered remodelling will result in a lower or higher BMD. Diet might influence BMD by affecting body weight and thus mechanical loading.

In addition, diet has the potential to modify the bone’s response to mechanical loading [9, 10], by either favourably or unfavourably affecting bone remodelling directly. It could be speculated that when mechanical loading is compromised due to weight loss, a diet-induced stimulation of remodelling will be more important to maintain a high BMD than when loading remains stable. Summarized, we hypothesize that body weight-induced changes in mechanical loading and diet-induced modifications in response to mechanical loading might interact in relation to BMD.

Hence, our primary aim was to identify dietary patterns that are associated with BMD in middle-aged and elderly subjects against a background of high dairy intake. Moreover, we explored whether the associations between dietary patterns and BMD might be influenced by body weight or changes in body weight over time.

## Methods

### Design

This study was embedded in the Rotterdam Study. Details on the objectives and design have been described previously [11]. In brief, Dutch subjects of 55 years and older living in the Ommoord district of Rotterdam, the Netherlands, were included in this prospective population-based cohort study. The Rotterdam Study has been approved by the institutional review board (Medical Ethics Committee) of the Erasmus Medical Centre and by the review board of The Netherlands Ministry of Health, Welfare and Sports.

### Baseline assessment of dietary intake

Baseline dietary intake of 170 food items was assessed by a trained dietician using a validated, semi-quantitative food frequency questionnaire (FFQ). The reliability of dietary intake was determined during this assessment by the dietician. For example, dietary data were considered as unreliable when patients experienced difficulties with recall or when they did not cooperate during the interview. The questionnaire was validated and adapted for use in the elderly [12, 13].The ability of the FFQ to rank subjects adequately according to their dietary intakes was demonstrated by a validation study (*n* = 80) comparing the FFQ to 15-day food records collected over a year to cover all seasons [14]. Pearson’s correlation coefficients of this comparison ranged from 0.4 to 0.8 for macro- and micronutrients after adjustment for sex, age, total energy intake and within-person variability in daily intakes.

### Identification of dietary patterns and assignment of pattern-adherence scores

All food items were categorized into 28 pre-defined food groups to reduce the complexity of dietary data. An overview of these food groups, which were based on similarities in product composition or culinary use, is shown in Supplemental Table 1. Next, dietary patterns were derived using Principal Component Analysis (PCA) on intake of these food groups in grams per day, unadjusted for total energy intake. We used Varimax rotation and Kaiser Normalization to obtain patterns with simpler structure [15] and optimal interpretability. Factor loadings, which reflect the standardized correlation between a food group and a dietary pattern, were used to characterize a pattern using a cut-off of 0.2, similar to comparable studies [16, 17]. Food groups with a factor loading >0.2 indicate a positive correlation with and <−0.2 a negative correlation with a specific pattern. Adherence to patterns with an Eigenvalue (a measure of explained variance) of >1.5 only was studied in relation to BMD. For each participant, pattern-adherence scores were constructed by summing up observed intakes of the pattern’s food groups weighted by the corresponding factor loading for each of the three dietary patterns separately.

### Longitudinal assessment of BMD

BMD of the femoral neck was measured by dual-energy X-ray absorptiometry (DXA) using a Lunar DPX-densitometer (Lunar Radiation Corp., Wadison, WI) at baseline and at three subsequent visits (1993–1995, 1997–1999 and 2002–2004). DXA scans were analysed with DPX-IQ (visit 1–3) and PRODIGY (visit 4) software. BMD values are expressed in g/cm^2^.

### Longitudinal assessment of anthropometrics

Body weight (kg) and height (cm) were assessed at the research centre repeatedly, during the same visits as at which the BMD measurements were assessed. Body weight was measured using a digital scale and body height was measured using a stadiometer, while subjects wore light clothing and no shoes.

### Assessment of covariates

The selection of covariates was based on previous studies investigating associations between dietary pattern-adherence and BMD [18–20]. A schematic overview of the data collection relevant to this study is shown in Supplemental Fig. 1.

#### Covariates assessed at baseline

Smoking was identified as “current” or “past” or “never”. Highest education and net household income were used as proxy for socioeconomic status (SES). Education was coded as “low” (primary education, primary + higher not completed, lower vocational and lower secondary education) or “high” (intermediate vocational, general secondary, higher vocational education & university). Household income was coded “above” or “below” the average of 2400 net Dutch Guilders (≈1600 euro) per month. Lower limb disability index, a combined index reflecting a subject’s ability to stand up, walk, climb and bend, was based on the Stanford Health Assessment Questionnaire [20]. Prevalent type 2 diabetes mellitus was determined as baseline serum glucose concentrations >11 mmol/l or use of glucose-lowering drugs. Prevalent CVD included prevalent coronary heart disease, heart failure, stroke and arterial fibrillation. Methods of data collection and definitions of cardiac outcomes in the Rotterdam Study have been described in detail elsewhere [21]. The use of serum-lipid-reducing agents and antihypertensive drugs was registered during the home interview by trained research assistants [22].

#### Covariates assessed at other visits

Use of hormone replacement therapy (HRT) in females was assessed at the 2nd visit and coded as “never” or “ever.” Physical activity was assessed on the 3rd visit, using the Zutphen Study Physical Activity Questionnaire including questions on walking, cycling, gardening, diverse sports, hobbies and housekeeping. Total time spent on physical activity was calculated by summing minutes per week for each type of activity [23–25]. Serum 25-hydroxyvitamin D (25(OH)D) was measured in a subgroup of participants (*n* = 3171) during the 3^rd^ visit to the research centre using radioimmunoassay’s (IDS Ltd, Boldon, UK, available at www.idsltd.com). The sensitivity of the test was 3 nmol/L which ranged from 4 to 400 nmol/L. Intra-assay accuracy was <8 %, and the inter-assay accuracy was <12 %.

### Status of body weight change; definitions of weight gain and weight loss

Weight loss and weight gain were defined as >5 % decrease or increase in baseline body weight during the full follow-up period (1989–2004). All other values were considered to indicate a stable body weight.

### Population of analysis

Of the full cohort of the Rotterdam (*n* = 7983), 1462 subjects did not attend the study centre and 271 were not offered an FFQ since they participated in the pilot phase of the Rotterdam Study only. Moreover, 122 participants were excluded due to suspected dementia, 2012 due to unreliable dietary intake data defined by the dietician and 481 were excluded for logistic reasons, leaving 5435 subjects with reliable intake data. Subjects were included for analysis when both reliable dietary intake data and at least one BMD measurement were available (*n* = 5144). Of these subjects, 4870 had measurements of BMD at baseline, 3682 at the second visit, 2561 at the third visit and 2305 at the fourth visit.

### Statistical analysis

#### Characteristics of the study population

Differences in characteristics between the tertiles of adherence to each dietary pattern were assessed using one-way Kruskal–Wallis tests for (non-normally distributed) continuous variables and *χ* ^2^ tests for categorical variables. These values are presented as median plus interquartile range (IQR) for continuous variables and as percentages for categorical variables.

We used the multiple imputation procedure for missing covariates using the Markov chain Monte Carlo method. Normally and non-normally distributed variables were predicted using predictive mean matching and binary or categorical variables using logistic regression.

#### Longitudinal associations between dietary pattern adherence and BMD

The association between adherence to the dietary patterns and BMD trajectories was studied using linear mixed modelling (LMM), a technique that takes the correlation between the repeated BMD measurements within each subject into account by including random effects in the model [26]. Specifically, we used a random intercept and slope (for time) model and assumed independent error terms. We used *Z* scores of adherence to each dietary pattern as exposure variables and sex-specific *Z* scores of BMD as the outcome. Despite using different densitometers in time we have shown in previous work, no cross-calibration is required [27]. The centre visit (1, 2, 3 or 4) was used as time variable and recoded as 0, 2, 6 and 10 years to adjust for differences in mean time interval between visits. Covariates were added to the model step wisely as independent variables to test for potential confounding and were kept in the multivariable model when they changed the regression coefficient of the associations between the dietary pattern adherence and BMD by >10 % [28].

Accordingly, three models were developed. The first was a basic model adjusted for age, sex and total energy intake and adherence to the other PCA-derived patterns (model 1). The second model was further adjusted for confounders and additionally included smoking, net household income, education, prevalent diabetes, physical activity and use of HRT (model 2). Since anthropometrics could be both confounders and intermediates in our analyses, we developed a third model that was further adjusted for body weight and height, which were measured repeatedly (model 3). Also, we studied longitudinal associations between dietary pattern adherences and body weight using model 2 with body weight (in kg) instead of BMD as the outcome, which was additionally adjusted for height. To assess whether adherences to dietary patterns were associated with trajectories of BMD, we tested the interaction with time by adding the product term of time x adherence score to the dietary pattern to model 3.

#### Influence of sex and changes in body weight

Effect modification by sex was tested by adding sex and the product term of sex *x* adherence score to the dietary pattern as independent variables to model 1.

We assumed that participants that experienced weight loss had more reduction of BMD and participants that experienced weight gain had less reduction of BMD over the follow-up period than those with stable weight. To test this assumption, we performed linear mixed models with BMD as the outcome and interaction term between weight loss or weight gain and time in models with body weight change (>5 % loss, stable (reference) or >5 % gain), age and sex. Only when our assumption was statistically confirmed, effect modification by body weight change was further evaluated.

Stratified analyses were only performed if the *P* for interaction was <0.10, using model 1. Stratified analyses for body weight were additionally adjusted for baseline body weight and height.

#### Sensitivity analyses

We performed two sensitivity analyses to compare the results of (1) our main analyses with and without using imputed covariates and (2) our stratified analyses using a more stringent cut-off to define weight gain or loss (±10 % instead of ±5 % change in body weight). LMM was performed using R statistical software version 3.2.1. (The R Foundation for Statistical Computing, Vienna, Austria). All other analyses were performed using SPSS software version 22 (IBM, Chicago, IL, USA).

## Results

### Dietary pattern identification

Three dietary patterns with an Eigenvalue of >1.5 were identified (scree plot in Supplemental Fig. 2), with a cumulative explained variance of 19 %, namely: (1) a “Traditional” dietary pattern characterized by high intake of potatoes, meat and fat and low intake of soy products; (2) a “Processed” dietary pattern characterized by high intake of processed meat, alcohol, mixed dishes like pizza, and low intake of fruit and yoghurt and (3) a “Health conscious” dietary pattern characterized by high intake of fruits and vegetables, poultry, fish and alcohol and low intake of sweets. A description and label of each pattern and the corresponding factor loadings per food group are shown in Table 1. None of the patterns has a factor loading for milk and milk products or cheese >0.2 or <−0.2. However, the factor loading for milk and milk products was close to this cut-off (−0.19) for the “Processed” pattern, which was low in yoghurt, another source of dairy products. However, despite a negative factor loading for yoghurt, also participants in the highest tertile of adherence to the “Processed” dietary pattern had relatively high intakes of total dairy products (2.3 serving per day vs. a median intake of 2.7 servings in the full study population), including milk, milk products and cheese as well as yoghurt.

**Table 1:** Factor loadings matrix and labels for the three dietary patterns that explained most of the variance in food group intake

Pattern	1	2	3
High factor loadings for	Meat, fat, potatoes, eggs	Processed meat, alcohol, mixed meals, eggs	Fruit, vegetables, poultry, fish, alcohol, eggs
Low factor loadings for	Soy products, mixed meals	Fruit, yoghurt	Sweets

Label	“Traditional”	“Processed”	“Health conscious”
Fruit and fruit products	−.036	**−.548**	**.219**
Vegetables and vegetable products	.182	−.187	**.240**
Pulses and legumes	−.046	−.010	−.110
Milk and milk products	.014	−.192	.054
Yoghurt	−.038	**−.506**	.114
Cheese products	−.037	.086	−.001
Soy products	**−.498**	.159	−.031
Refined grain products	.005	.170	.082
Whole grain products	.063	.011	−.052
Soft drinks and lemonades	.097	−.082	.149
Eggs	**.280**	**.258**	**.257**
Unprocessed meat	**.641**	.086	−.076
Processed meat	**.520**	**.451**	.054
Poultry	−.023	−.022	**.494**
Fatty fish	−.071	.137	**.524**
Lean and battered fish	−.029	−.150	**.629**
Shell fish	.032	−.024	**.326**
Savoury snacks	.015	−.073	−.006
Sweets	.131	−.177	**−.211**
Nuts and seeds	−.007	.089	.051
Vegetable oils and fats	**.296**	.072	.006
Animal fats	**.243**	.092	−.090
Coffee tea and water	.027	−.135	.081
Alcoholic drinks	.154	**.558**	**.202**
Mixed meals^a^	**−.208**	**.372**	.117
Soups and sauces	.089	.077	.192
Potatoes	**.582**	.095	−.185
Porridges	−.064	−.008	.007

Extraction method: principal component analysis, Rotation method: varimax with Kaiser normalization. Rotation converged in 17 iterations

Factor loadings represent the standardized correlations between the food groups and the dietary patterns

Factor loadings >0.2 or <−0.2 are in bold and were used to label the dietary patterns

Bami and Nasi are traditional Indonesian dishes with meat, vegetables and rice (Nasi) or pasta (Bami) and could reflect either home-made or take-away food)

^a^Mixed meals included Pizza, Nasi and Bami Goreng

### Study population for investigating associations between pattern adherence and BMD

The median total dairy intake of our study population was 2.7 servings per day and was mainly determined by daily consumptions of milk and milk products (1.4 servings) and cheese products (0.9 servings, Supplemental Table 2).

Characteristics of subjects in each tertile of adherence to the three dietary patterns are shown in Table 2. Briefly, subjects with high adherence to the “Traditional” and “Processed” patterns were more often males (59 vs. 26 and 62 vs. 24 % for the highest vs. the lowest tertile, respectively (*P* for difference <0.001). Smoking was more prevalent in subjects with high adherence to the “Processed” pattern. Females with high adherence to the “Health conscious” pattern were more likely to have used HRT. No clear differences in age, physical activity or indicators of SES were observed. Calcium intake was constant over the tertiles of adherence to the “Traditional” pattern (*P* for difference = 0.59), and time spent on vigorous physical activity was constant over the tertiles of the “Processed” pattern (*P* for difference = 0.15). Between baseline and the 4th visit, mean BMD slightly decreased in females (1.2 %), but not in males. At the same time, mean body weight increased in both males (+3.4 %) and females (+2.3 %). Median intake of food groups in the lowest and highest tertile is shown in Supplemental Table 2.

**Table 2:** Characteristics of participants of the Rotterdam Study (*N* = 5435) per tertile of adherence to the “Traditional”, “Processed “or “Health conscious” dietary pattern

	“Traditional” pattern			“Processed” pattern			“Health conscious” pattern		
	1st tertile	2nd tertile	3rd tertile	1st tertile	2nd tertile	3rd tertile	1st tertile	2nd tertile	3rd tertile
Age (years)^a^	67 (61–74)	67 (62–74)	66 (61–72)	68 (62–74)	68 (62–74)	66 (61–72)	68 (63–75)	66 (61–73)	67 (61–72)
Total energy intake (kcal/d)^a^	1684 (1453–1987)	1877 (1616–2161)	2210 (1922–2538)	1886 (1602–2233)	1861 (1581, 2191)	2027 (1687, 2351)	1929 (1616–2262)	1887 (1599–2208)	1955 (1634–2304)
Physical activity (h/day)^a^	5.9 (4.3–7.9)	5.6 (4.0–7.4)	5.9 (4.4–8.0)	6.0 (4.3, 8.0)	5.7 (4.0, 7.4)	5.7 (4.2, 7.8)	5.6 (3.9–7.6)	6.0 (4.3–7.8)	5.9 (4.4–7.9)
Of which vigorous (h/week)^a^	3.3 (1.3–6.5)	3.1 (1.0–6.7)	3.5 (1.0–7.0)	3.2 (1.5–7.3)	3.3 (1.0, 6.5)	3.5 (1.0–7.0)	3.0 (1.0–6.3)	3.5 (1.3–7.0)	3.6 (1.5–7.2)
Dutch healthy diet index^a,b^	52 (45–59)	49 (43, 56)	44 (38–51)	53(46–59)	49 (42–55)	44 (37, 50)	44 (37–51)	49(42–56)	52 (45–58)
Calcium intake (mg/day)^a^	1090 (869, 1331)	1075 (863, 1304)	1066 (857, 1331)	1208 (997, 1475)	1053 (858, 1286)	968 (758, 1203)	1017 (814–1260)	1077 (870–1319)	1141 (899–1390)
25 (OH) D3 (nmol/l)^a,c^	41 (27–57)	46 (30–65)	47 (31–69)	43 (28–62)	43 (29, 62)	48 (32, 69)	41(26–60)	46(30–64)	47 (32–68)
BMD at 1st visit (mg/cm^2^)^a,f^	0.84 (0.75, 0.94)	0.85 (0.77, 0.94)	0.89 (0.79, 0.99)	0.85 (0.76–0.94)	0.86 (0.77–0.95)	0.88 (0.78–0.97)	0.85 (0.76–0.94)	0.86 (0.77–0.96)	0.88 (0.78–0.97)
BMD at 2nd visit (mg/cm^2^)^a,f^	0.84 (0.74, 0.94)	0.85 (0.76, 0.95)	0.89 (0.79, 0.98)	0.85 (0.75–0.95)	0.85 (0.76–0.95)	0.88 (0.78–0.98)	0.85 (0.74–0.94)	0.86 (0.76–0.96)	0.88 (0.78–0.97)
BMD at 3rd visit (mg/cm^2^)^a,f^	0.84 (0.74, 0.93)	0.86 (0.76, 0.96)	0.89 (0.79, 0.99)	0.85 (0.75–0.95)	0.85 (0.76–0.95)	0.88 (0.78–0.98)	0.84 (0.74–0.95)	0.86 (0.77–0.96)	0.88 (0.79–0.97)
BMD at 4th visit (mg/cm^2^)^a,f^	0.84 (0.74, 0.92)	0.85 (0.76, 0.94)	0.88 (0.78, 0.98)	0.84 (0.75–0.94)	0.85 (0.76–0.94)	0.86 (0.76–0.93)	0.83 (0.74–0.92)	0.85 (0.76–0.95)	0.87 (0.77–0.96)
Body weight at 1st visit (kg)^a^	70 (63, 78)	73 (66, 80)	76 (69, 84)	72 (65–81)	73 (65–81)	75 (67–83)	72 (65–80)	72 (65–80)	74 (67–83)
Body weight at 2nd visit (kg)^a^	70 (63, 78)	74 (66, 81)	77 (69, 85)	72 (65–81)	73 (64–80)	76 (68–83)	72 (65–80)	73 (65–81)	75 (68–83)
Body weight at 3rd visit (kg)^a^	71 (62, 79)	74 (66, 81)	77 (69, 85)	73 (65–83)	73 (65–81)	77 (68–84)	73 (64–80)	73 (65–81)	75 (68–83)
Body weight at 4th visit (kg)^a^	72 (63, 81)	75 (67, 83)	78 (70, 87)	73 (65–82)	4 (66–83)	77 (68–86)	74 (65–82)	74 (66–83)	76 (69–85)
Baseline body height (cm)^a^	164 (159, 171)	166 (160, 173)	171 (164, 177)	164 (160–171)	166 (160–173)	171 (164–177)	167 (160–174)	166 (161–173)	167 (161–174)
Sex (% males)	26	38	59	24	36	62	58	61	58
Body weight change (% loss/gain)^d^	15/36	17/34	18/29	17/35	17/32	16/31	19/29	16/33	15/35
Prevalent osteoporosis (%)	14	12	8	11	13	10	14	11	9
Prevalent type 2 diabetes (%)	9	9	11	9	9	11	10	9	10
Prevalent CVD (%)	12	13	13	11	14	12	13	12	12
High education (%)	36	36	38	32	35	44	33	37	40
High income (% >1600 euro/mo)	47	51	54	48	48	56	46	51	55
Current smokers (%)	19	20	28	14	22	34	24	22	23
Current or past HRT use (%)^e^	9	10	8	9	9	8	8	8	11
Lipid lowering drug use (%)	3	2	2	3	3	2	2	3	3
Antihypertensive drug use (%)	12	13	13	12	14	12	13	13	13
Lower limb disabled (%)	20	18	15	20	19	15	20	16	17

*CVD* cardiovascular disease, *HRT* Hormone replacement therapy

^a^Median (interquartile range)

^b^The Dutch Healthy Diet Index reflects adherence to the Dutch guidelines for a healthy diet and included information on intake of vegetables, fruit, fibre, fish, saturated fatty acids, trans fatty acids, acidic drinks and foods, sodium and alcohol (van der Lee 2012)

^c^Measured at the 3rd visit

^d^Weight loss or gain is defined as >5 % reduction or increase in body weight

^e^Females only

^f^Sample sizes of BMD were *N* = 4870 for visit 1, *n* = 3682 for visit 2, *n* = 2561 for visit 3 and *n* = 2305 for visit 4

### Associations between adherence to identified dietary patterns and BMD

Regression coefficients and 95 % CI of the associations between adherence to all dietary patterns and repeatedly measured BMD (standardized, all in *Z* scores) are shown in Table 3. After adjustment for potential confounders (model 2), adherence to the “Traditional” pattern and the “Health conscious” pattern were significantly associated with higher BMD (*β*: 0.06; 95 % CI 0.03, 0.09 for “Traditional” and *β*: 0.06; 95 % CI 0.03, 0.08 for “Health conscious” dietary pattern). In contrast, adherence to the “Processed” pattern was significantly associated with lower BMD (*β*: −0.03; 95 % CI −0.06, −0.01).

**Table 3:** Dietary pattern adherence and BMD of the femoral neck, obtained using linear mixed modelling with random intercept and slope

Adherence to the:	Model 1^1^	Model 2^1^	Model 3^1^	*P* for interaction with time^2^
“Traditional” pattern	**0.05** (**0.02**, **0.08**)	**0.06** (**0.03**, **0.09**)	0.01 (−0.01, 0.04)	0.48
“Processed” pattern	−**0.05** (**−0.08**, **−0.02**)	**−0.03** (**−0.06**, **−0.01**)	**−0.03** (**−0.06**, **−0.00**)	0.99
“Health conscious” pattern	**0.06** (**0.04**, **0.09**)	**0.06** (**0.03**, **0.08**)	**0.04** (**0.02**, **0.07**)	**0.01**

Model 1: Adjusted for age, sex, total energy intake and adherence to other dietary patterns (basic model)

Model 2: Model 1 + additional adjustment for SES, smoking, prevalent T2DM at baseline, total physical activity and use of lipid lowering drugs

Addition of lower limb disability, prevalent CVD at baseline, use of HRT or antihypertensive drugs and plasma vitamin D did not change the effect estimate by ≥10 %

Model 3: Model 2 + additional adjustment for body weight and height

*BMD* bone mineral density, *CVD* Cardiovascular disease, *HRT* Hormone replacement therapy, *SD* standard deviation

In bold *P* value <0.05

^1^Regression coefficients (95 % confidence intervals) of the fixed effects. Regression coefficients represent differences in BMD (in sex-specific *Z* scores) for each SD of increase in dietary pattern adherence

^2^The *P* value for interaction with time was tested using model 1, to study the association between dietary pattern adherence and BMD trajectories. A significant *P* for interaction reflects that high adherence to a specific dietary pattern is associated with less decline of BMD over time

The *P* value for interaction with time was only significant for the “Health conscious” pattern (*P* = 0.01), which reflects that high adherence to this dietary pattern is associated with less decline of BMD over time.

### Influence of body weight and height or changes in body weight status

The “Traditional” and “Health conscious” patterns were associated with high body weight [(*β*: 1.79; 95 % CI 1.50, 2.09) and (*β*: 0.84; 95 % CI 0.60, 1.11) kg per *Z* score of pattern adherence]. In contrast, the “Processed” pattern was not significantly associated with body weight. After additional adjustment for body weight and height in the analyses of dietary patterns and BMD (Table 3, model 3), a significant association between adherence to the “Health conscious” pattern and high BMD remained. However, the magnitude of the effect was diluted (*β*: 0.04; 95 % CI 0.02, 0.07 in model 3 vs. *β*: 0.06; 95 % CI 0.03, 0.08 in model 2). In contrast, the significant association between adherence to the “Traditional” pattern and BMD was lost after adjustment for body weight and height, whereas the association between adherence to the “Processed” pattern and low BMD was not affected by additional adjustment for body weight and height.

**Fig. 1:**
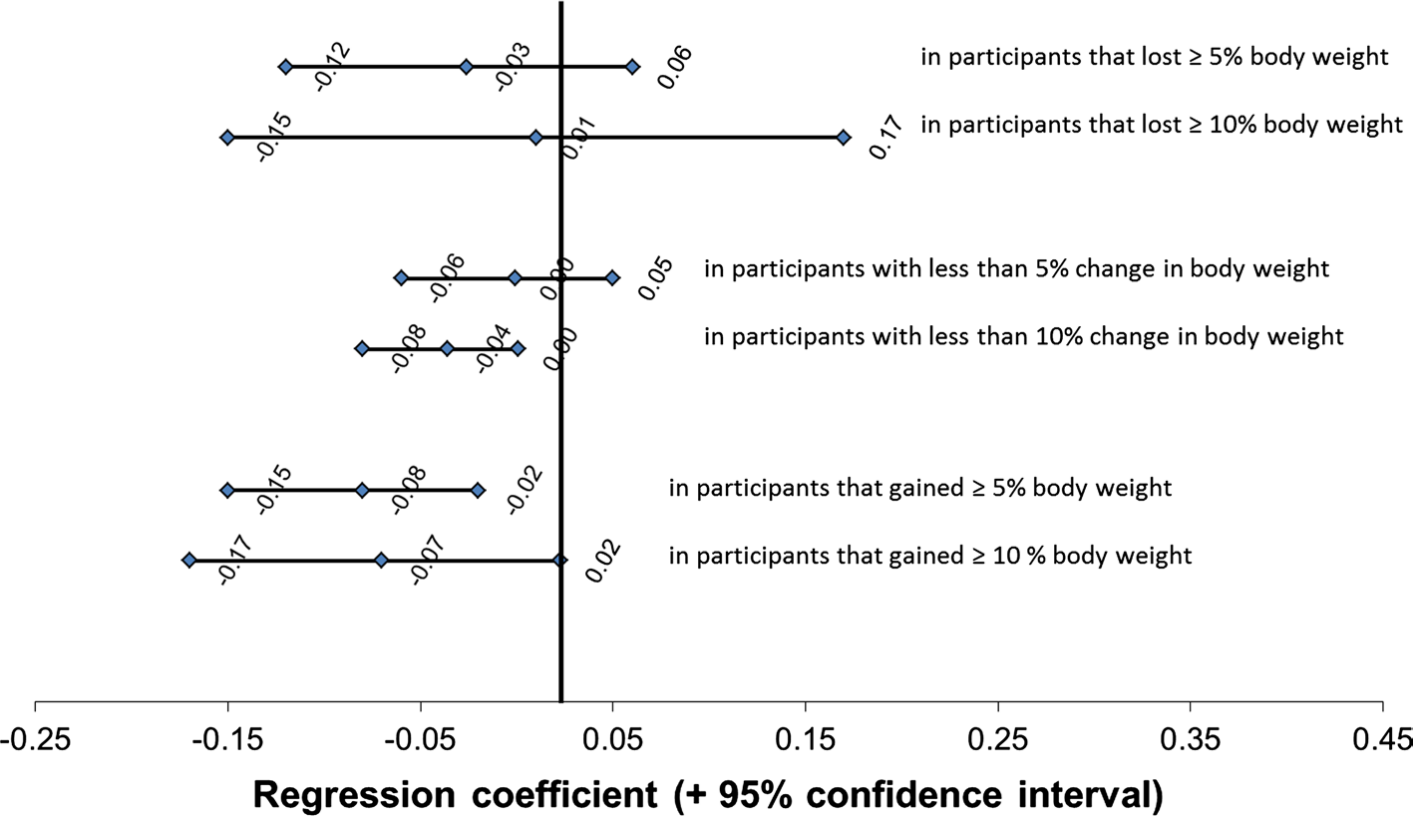
Associations between adherence to the “Processed” dietary pattern and BMD of the femoral neck, in strata of body weight change between baseline and visit 4 (*n* = 2532). ^1^Regression coefficients and 95 % confidence intervals of the fixed effects. Regression coefficients represent differences in BMD (in sex-specific *Z* scores) for each Z-score of increase in adherence to the “Processed” dietary pattern using a cut-off of 5 % (*solid line*) or 10 % (*dashed line*) change in body weight to define weight loss or weight gain. Models are adjusted for age, sex, initial body weight and height, total energy intake and adherence to the other two dietary patterns. *BMD* bone mineral density, *SD* standard deviation

We observed significant interaction between weight loss and weight gain with time in relation to BMD. This substantiates our assumption that participants that lost weight experienced more reduction of BMD and participants that gained weight experienced less reduction of BMD over the follow-up period that participants with stable body weight. Interaction with body weight change was only observed for adherence to the “Processed” pattern (*P* for interaction = 0.06), but not for both other patterns (*P* for interactions >0.55). Data may suggest a stronger association between adherence to the “Processed” pattern and low BMD in subjects that experienced ≥5 % weight gain (*β*: −0.07; 95 % CI −0.17, −0.02) than in those with ≥5 % weight loss (*β*: −0.03; 95 % CI −0.12, 0.06, Fig. 1). No interaction between adherence to any dietary pattern with sex in relation to BMD was observed (*P* all interactions > 0.60).

### Sensitivity analyses

Multiple imputation of missing covariates did not markedly affect the effect estimates of adherence to all dietary patterns in relation to BMD (data not shown). Also, the use of a more stringent cut-off to define weight gain or loss (±10 % instead of ±5 % change in body weight) did not change the results of our stratified analysis (Fig. 1).

## Discussion

### Summary of main findings

In this Dutch population of middle-aged and elderly subjects, we identified two dietary patterns that were associated with higher BMD; a “Traditional” pattern (characterized by high intakes of potatoes, meat and fat) and a “Health conscious” dietary pattern (characterized by high intakes of fruits, vegetables, poultry, fish and alcohol). In contrast, adherence to a “Processed” pattern (characterized by high intakes of processed meat, mixed meals and alcohol) was associated with low BMD. The associations between adherence to the “Traditional” pattern and BMD were explained, at least partly, by differences in body weight and height.

### Comparison with published dietary pattern analyses

The observed associations are to some extent similar to those reported in previous studies. Data from the Canadian Multicentre Osteoporosis Study (CAMOS) suggest that a “nutrient-dense” diet high in fruit, vegetables, whole grains and fish was associated with high BMD [*β*: 0.01 (95 % CI 0.00, 0.02) in g/cm^2^ per *Z* score of pattern adherence] after adjustment for BMI [17]. This dietary pattern was similar to the “health conscious” pattern that we identified in our study population. The combined intake of fruits, vegetables and fish was also shown to be associated with high BMD in Japanese farmwomen, when consumed in a pattern with soy products [29]. The existing Mediterranean Diet Score (MDS), developed by Trichopoulou et al. [30], was shown to be associated with high BMD [31]. Studies on the MDS and fracture risk showed both unfavourable [2] and favourable [32] results. The MDS reflects high intake of cereals, legumes, fruits & nuts, vegetables, oils and fish and low intake of dairy and meat products. Although none of the dietary patterns that were defined in our population exactly reflects the Mediterranean diet, it could be argued that it has similarities to our “Health conscious” pattern, due to its high factor loadings for fruits, vegetables and fish.

The “Processed” pattern which was high in processed meat, alcohol and mixed meals and low in yoghurt was associated with low BMD. The association of patterns high in meat and unhealthful, energy-dense food products with low BMD was also observed in several other populations, including Iranian women [33] and Canadian men (0.009 g/cm^2^ decrease per *Z* score of pattern adherence) and women (0.004 g/cm^2^ decrease per *Z* score of pattern adherence) [17].

### Explanation of our results and potential mechanisms

To identify important dietary components underlying the observed associations between the “Processed” and “Health conscious” pattern and BMD, it is not only relevant to study the factor loadings of our food groups to these patterns, but also the absolute intakes of these food groups. For example, the factor loading of mixed meals for the “Processed” pattern (0.37) indicates a strong correlation, but the intake of mixed meals in the highest tertile of adherence to the “Processed” pattern is <1 serving per month (Supplemental Table 2). It is therefore unlikely that these individual food groups explain our results. More plausibly, the intake of fruits, vegetables or fish could have contributed in either an additive or synergistic manner to the observed relation with high BMD. Vitamin D intake from sources such as fatty fish could explain the relation with high BMD, as it is well established that vitamin D is needed for calcium uptake by the intestine and important for bone health [34]. Fruits and vegetables contain a variety of nutrients that might explain positive associations of a diet with high factor loadings for these food groups, such as magnesium, vitamin C, carotenoids and potassium [35]. Magnesium might contribute to healthy bone remodelling [36] via its favourable impact on osteoblastic and osteoclastic activity and vitamin C and carotenoids might explain the association via antioxidant-related mechanisms [1]). Moreover, poultry and fish, rather than red meat, might be sources of protein that are beneficial for bone remodelling. Negative associations of red and organ meat, but not poultry, with bone outcomes were also shown in Chinese elderly [37], a finding which may be explained by differences in fat or amino acid content or quality. Also, a potential interplay between calcium, sodium, magnesium and phosphorus could play a role. For example, an excess intake of phosphorus, especially from processed food products as found in the “Processed” pattern has been suggested previously to disrupt hormonal regulation of calcium and vitamin D, thereby leading to low BMD [38].

There is general consensus that body weight is a main determinant of BMD [39], as it influences mechanical loading of the weight-bearing bones. In our analysis, we took two approaches to investigate the influence of body weight on the associations between dietary pattern adherence and BMD. First, we built an additional model adjusted for repeatedly measured body weight and height, and second, we tested for interaction between dietary pattern adherence and status of body weight change in relation to BMD. The “Traditional” and the “Health Conscious” dietary pattern both showed positive associations with BMD, despite their highly different food group composition (potatoes, meat and fat vs. fruits, vegetables, poultry and fish). Since the association between the “Traditional” pattern and BMD was mainly explained by differences in body weight and height, we can hypothesize that adherence to the “Traditional” dietary pattern influences BMD by increasing body weight and consequently mechanical loading. In contrast, the association between adherence to the “Health Conscious” dietary pattern and BMD was independent of body weight and height. We therefore hypothesize that adherence to this dietary patterns might have influenced BMD by influencing the bone’s response to mechanical loading, rather than loading itself (in line with the Mechanostat Theory proposed by Frost [9]). We found no evidence that associations between adherence to any of the dietary patterns and BMD were different for people that lost or gained body weight than for those with stable body weight.

### Strengths and limitations

Our study has several strengths. First, we had repeated measurements of BMD and anthropometrics, allowing longitudinal analyses on dietary patterns and BMD with precise adjustment for body weight and height. Second, we had a large sample which included both males and females. Third, to our knowledge, we are the first to investigate the relationship between dietary patterns and BMD against a background of high dairy intake (median intakes 19 servings per week). We also recognize some limitations. Dietary intake, assessed using an FFQ, was self-administered and therefore susceptible to measurement error. However, the ability to properly rank subjects into categories of low to high intake was established in a validation study that compared the FFQ to a 24-h recall in a random sample of The Rotterdam Study [14]. Also, dietary intake was assessed at baseline only. Changes in dietary behaviour over time might have affected the results. However, it has been shown in a comparable cohort that ranking of individuals is fairly similar when using a single FFQ measurement than when using repeated measurements [40]. Participants with dietary intake data were slightly younger, more often non-smokers, less likely to have prevalent type 2 diabetes and more likely to use hormone replacement therapy than participants of the full cohort (*n* = 7983). It could therefore be stated that our study population was slightly healthier than our full cohort and was therefore more likely to adhere to a healthy diet and to have high BMD. This does not necessarily imply that our association under study cannot be translated to the full cohort and general population due to selective participation. The latter assumption was supported by recent findings of Winding et al. 2014 in a Danish youth cohort [41]. Hence, we believe that our results are still valid. Despite our effort to adjust for a number of confounders, residual confounding related to an overall healthy lifestyle might still be present. Also the single measurement of physical activity and plasma vitamin D only at the third visit may have led to residual confounding by physical activity and vitamin D levels.

The use of a PCA to determine dietary patterns has some methodological limitations. Although the “a posteriori” nature of the patterns identified provides a realistic reflection of dietary patterns in our study population, it does not necessarily provide the most optimal dietary pattern (3) in relation to BMD and may affect the external validity of the results. In addition, several decisions such as the clustering of food items into groups and extraction of the patterns from the PCA are to some extent subjective to the investigator and may affect the final dietary patterns that are analysed. The three patterns identified in this study explain 20 % of the overall variance, which is similar to some [18] but lower than other studies [33, 42] investigating dietary patterns in relation to bone, This shows the complexity of efficiently using dietary intake data and may affect the external validity of our results. Lastly, data were only available on BMD of the femoral neck, and not of the spine. Some studies have shown that dietary patterns were associated with BMD of the lumbar spine, but not of the femoral neck [33, 42], so we might have not been able to detect additional associations between our dietary patterns and spinal BMD.

### Implications, recommendations and future perspectives

Contributing to the development of food-based dietary guidelines, a systematic review on the relationship between dietary patterns and health outcomes has been published by the United States Department of Agriculture. These food-based dietary guidelines were based mainly on studies on overweight and underweight, cardiovascular disease and type 2 diabetes. However, some studies on osteoporosis have been included [43].

With that in mind, we believe that our study could contribute to further improvement of food-based dietary guidelines in relation to bone health. Our results indicate that, against a background of high dairy intake, different dietary patterns may influence BMD. Although food groups such as fruits and vegetables are included in many dietary guidelines [44], specific advice on high consumption of poultry, eggs and limited consumption of processed meat is not always included.

In addition, it would be worthwhile to investigate further the effects of different food groups beyond calcium-rich foods, such as dairy, on bone mineralization. It would be beneficial to investigate the effects of the different food groups at both the population level and the mechanistic level. Another field of research could focus on the role of fat quality and potential differences in effects between diets rich in meat versus poultry and fish.

## Conclusion

Against a background of high dairy intake in this population, a “Health conscious” dietary pattern, characterized by high intake of fruit, vegetables, fish and poultry, may have benefits for BMD independent of anthropometrics. In contrast, adherence to a “Processed” dietary pattern characterized by high intake of processed meat, mixed meals and alcohol may pose a risk for low BMD.

## Electronic supplementary material

Below is the link to the electronic supplementary material.

Supplementary material 1 (DOCX 165 kb)
