# Supplementary material for: Dietary patterns in an elderly population and their relation with bone mineral density: the Rotterdam Study
Source: Eur J Nutr. 2016 Aug 24;57(1):61–73. doi: 10.1007/s00394-016-1297-7 (PMC5847075; doi:10.1007/s00394-016-1297-7)
Supplement: Supplementary file 1 — Supplementary material 1 (DOCX 165 kb) [file 394_2016_1297_MOESM1_ESM.docx]

**ONLINE SUPPLEMENTAL MATERIALS**

Supplemental Table 1: Pre-defined food groups for Principal Component Analysis

| **Food group** | **Food items included** |
| --- | --- |
| Fruit (products) | Fresh fruits, dried fruits, fruit cocktail in syrup, fruit juices (unsweetened apple juice, grapefruit juice and orange juice) |
| Vegetable (products) | Vegetables raw or boiled, gherkins, vegetable juices, mushrooms |
| Pulses & legumes | Beans broad, Beans white or brown, Pea marrow fat legumes |
| Milk (products) | Buttermilk, (semi) skimmed and whole milk chocolate milk, coffee creamer, custards and puddings, dairy based ice cream and whipping cream |
| Yoghurts | Low fat, half fat and full fat yoghurt, plain and with fruits, fromage fraise |
| Cheese (products) | Cheese and cheese spreads varying in fat content (>48, >40 and >20% fat) and sodium content |
| Soy products | Soya chunks, Tahoe soya curd |
| Refined grains | Bread white, Currant bread, Macaroni cooked, Rice white boiled, Rusk Dutch |
| Whole grains | Bread brown wheat/ whole meal, crisp bread, Muesli, Rice brown Rye bread, Wheat bran & germ |
| Potatoes | Potatoes, boiled |
| Soft drinks & lemonades | Cola soft drink with/ without caffeine, fruit drink several flavors, carbonated mineral water |
| Eggs | Eggs, boiled |
| Unprocessed meat | Beef raw, Hamburger, Horsemeat, Lamb, Liver chicken/ ox/ pork, Mutton, Pork, Veal |
| Processed meat | Bacon, Beef salted and smoke dried, Corned beef, Croquette meat ragout deep  fat fried, ham, sausages and salami |
| Poultry | Chicken fillet with and without skin |
| Fatty fish | Eel, Fish, 2-10 g fat and > 10 g fat raw, Herring, Mackerel, Plaice Salmon Sardines/pilchards (fresh and canned) |
| Lean and battered fish | Cod, Fish fingers, Fish lean 0-2 g fat raw, Haddock fillet in batter fried |
| Shell fish | Mussels boiled, Shrimps, peeled, boiled |
| Savoury snacks | Biscuit salted average, Crisps, Liquorice Dutch type salted |
| Nuts and seeds | Nuts mixed unsalted, Peanut butter, Peanuts coated, Peanuts salted, Peanuts unsalted, Linseed |
| Mixed meals | Bami Goreng, Nasi, Pizza  *(Bami and Nasi are traditional Indonesian dishes with meat, vegetables and*  *rice (Nasi) or pasta (Bami) and could reflect either home-made or take-away*  *food)* |
| Soups and sauces | Salad dressings, salad creams |
| Sweets | Sweet bread toppings, pie, biscuits, cake, chocolate bars, spiced honey cake, gateau, honey, popsicle ice cream, candy, pancakes, praline and sugar |
| Coffee tea and water | Coffee or tea prepared, water |
| Vegetable oils and fats | Cooking or frying fat 0-50 mg cholesterol, Margarine, Oils (corn germ, olive, peanut, safflower, soy and sunflower) |
| Animal fats | Butter unsalted, Frying fat > 50 mg cholesterol |
| Alcoholic drinks | Beer pilsner, Gin young Dutch, Sherry, Wine |
| Porridges | Porridge buttermilk with wheat flour paste, Porridge oatmeal, Porridge rice pasteurized |

Supplemental Table 2: Median intake of food groups in lowest and highest tertile of adherence to each pattern

|  | **“Traditional”** | | | **“Processed”** | | | | **“Health conscious”** | | | **Full study population** |
| --- | --- | --- | --- | --- | --- | --- | --- | --- | --- | --- | --- |
|  | **1^st^ tertile** | **3^rd^ tertile** | | **1^st^ tertile** | | **3^rd^ tertile** | | **1^st^ tertile** | | **3^rd^ tertile** |  |
|  | **Intake of food groups that are frequently consumed in servings per day** | | | | | | | | | |  |
| Potatoes | **0.5** | | **0.9** | | 0.7 | 0.7 | | 0.8 | | 0.7 | 0.8 |
| Fruit | 2.1 | | 2.3 | | **3.0** | **1.3** | | **1.8** | | **2.4** | 2.0 |
| Vegetables | 5.8 | | 7.8 | | 6.6 | 5.8 | | **5.2^1^** | | **7.2** | 6.1 |
| Sweets | 3.5 | | 4.0 | | 3.8 | 2.5 | | **4.0** | | **2.8** | 3.6 |
| Vegetable oils and fats | **1.8** | | **2.7** | | 1.9 | 2.2 | | 1.9 | | 2.0 | 2.3 |
| Milk-and milk products | 1.4 | | 1.3 | | 1.6 | 1.1 | | 1.3 | | 1.4 | 1.3 |
| Cheese products | 1.0 | | 0.9 | | 0.9 | 1.0 | | 0.9 | | 0.9 | 0.9 |
| Yoghurt | 0.2 | | 0.2 | | **0.5** | **0.0** | | 0.1 | | 0.3 | 0.2 |
| *Total dairy intake^2^* | 2.8 | | 2.6 | | 3.0 | 2.3 | | 2.6 | | 2.8 | 2.7 |
|  | **Intake of food groups that are frequently consumed in servings per week** | | | | | | | | | |  |
| Eggs | **1.0** | | **2.0** | | **1.5** | **2.0** | | **1.0** | | **2.0** | 2.0 |
| Alcoholic drinks | 0.2 | | 0.5 | | **0.2** | **1.6** | | **0.2** | | **0.6** | 1.0 |
| Unprocessed meat | **1.6** | | **3.4** | | 2.3 | 2.4 | | 2.4 | | 2.1 | 2.5 |
| Processed meat | **0.6** | | **1.4** | | **0.7** | **1.2** | | 0.8 | | 0.9 | 1.1 |
|  | **Intake of food groups that are rarely consumed in servings per month** | | | | | | | | | |  |
| Poultry | 1.4 | | 1.7 | | 1.9 | | 1.3 | **0.6** | **2.7** | | 1.7 |
| Fatty fish | 0.0 | | 0.0 | | 0.0 | | 0.0 | **0.0** | **0.6** | | 0.0 |
| Lean /battered fish | 1.3 | | 1.3 | | 1.6 | | 0.6 | **0.0** | **3.9** | | 1.3 |
| Animal fats | **0.0** | | **12.0** | | 0.0 | | 1.7 | **0.0** | **0.0** | | 0.0 |
| Mixed meals | 0.0 | | 0.0 | | **0.0** | | **0.7** | 0.0 | 0.0 | | 0..0 |
| Tofu | **0.5** | | **0.0** | | 0.0 | | 0.4 | 0.0 | 0.0 | | 0.0 |

Product groups with factor loadings >0.2 or < 0.2 for a specific dietary pattern are indicated in **bold**. ^1^: A serving size equals 30 grams of vegetables (Donder-Engelen and van der Heijden, Maten, Gewichten en codenummers/ Measures, Weights and Codes (2003) Wageningen University, The Netherlands-CD Rom) ^2^: Summed intake of milk-and milk products, yoghurt and cheese products.

**SUPPLEMENTAL FIGURES**

**
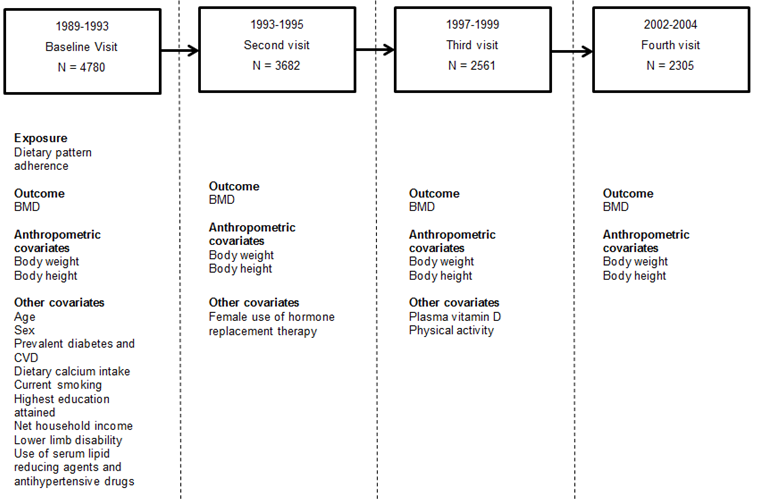
**

Supplemental Figure 1: Assessment of exposure, outcome and covariates

**
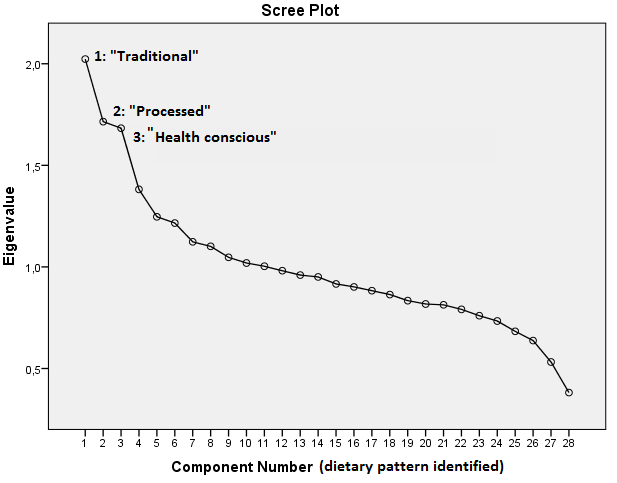
**

| **Total Variance Explained** |
| --- |

| Component | Initial Eigenvalues | | | Rotation Sums of Squared Loadings | | |
| --- | --- | --- | --- | --- | --- | --- |
|  | Total | % of Variance | Cumulative % | Total | % of Variance | Cumulative % |
| **1 “Traditional”** | 2.023 | 7.226 | 7.226 | 1.651 | 5.895 | 5.895 |
| **2 “Processed”** | 1.715 | 6.126 | 13.352 | 1.526 | 5.450 | 11.345 |
| **3 “Health conscious”** | 1.683 | 6.010 | 19.362 | 1.449 | 5.174 | 16.519 |

Supplemental Figure 1: Scree plot showing the Eigenvalue (y-axis), a measure of explained variance, for each dietary pattern (x-axis) identified by principal component analysis.

Using a cut-off for the Eigenvalue of 1.5, we have selected three dietary patterns. Adherences to these three dietary patterns were studied in relation to bone mineral density.
